# Resolving Conflicts Between People and Over Time in the Transformation Toward Sustainability: A Framework of Interdependent Conflicts

**DOI:** 10.3389/fpsyg.2021.623757

**Published:** 2021-04-15

**Authors:** Johann M. Majer, Matthias Barth, Hong Zhang, Marie van Treek, Roman Trötschel

**Affiliations:** ^1^Department of Social, Organizational, and Political Psychology, Faculty of Education, Institute of Psychology, Leuphana University Lüneburg, Lüneburg, Germany; ^2^Education for Sustainable Development, Faculty of Sustainability, Institute of Sustainable Development and Learning, Leuphana University Lüneburg, Lüneburg, Germany

**Keywords:** conflict, transformation, sustainability, negotiation, intrapersonal conflict, intergenerational conflict, transition management

## Abstract

Transformative and mutually beneficial solutions require decision-makers to reconcile present- and future interests (i.e., intrapersonal conflicts over time) and to align them with those of other decision-makers (i.e., interpersonal conflicts between people). Despite the natural co-occurrence of intrapersonal and interpersonal conflicts in the transformation toward sustainability, both types of conflicts have been studied predominantly in isolation. In this conceptual article, we breathe new life into the traditional dialog between individual decision-making and negotiation research and address critical psychological barriers to the transformation toward sustainability. In particular, we argue that research on intrapersonal and interpersonal conflicts should be tightly integrated to provide a richer understanding of the interplay between these conflicts. We propose a novel, unifying framework of interdependent conflicts that systematically structures this interplay, and we analyze how complex interdependencies between the social (i.e., conflict between decision-makers) and temporal (i.e., conflict within a decision-maker) dimensions pose fundamental psychological barriers to mutually beneficial solutions. Since challenges to conflict resolution in the transformation toward sustainability emerge not only between individual decision-makers but also frequently between groups of decision-makers, we scale the framework up to the level of social groups and thereby provide an interdependent-conflicts perspective on the interplay between intra- and intergenerational conflicts. Overall, we propose simple, testable propositions, identify intervention approaches, and apply them to transition management. By analyzing the challenges faced by negotiating parties during interdependent conflicts and highlighting potential intervention approaches, we contribute to the transformation toward sustainability. Finally, we discuss implications of the framework and point to avenues for future research.

## Introduction

Human civilization stands at a crossroads. Avoiding a decline of the human species and ensuring its long-term survival requires scaling up human cooperation at all levels, from individual to global (Dreber and Nowak, 2008; Ostrom, 2009; Dannenberg and Barrett, 2018). Sustainability issues such as climatic change, biodiversity loss, and resource depletion can result in a conflict of interests between individuals, groups, organizations, and nations (Hsiang et al., 2013; Mach et al., 2019). These challenges inevitably require collaborative decision-making processes (i.e., negotiations) to coordinate different interests and reach conflict solutions (Barrett and Dannenberg, 2012; Ehrlich and Ehrlich, 2013). Negotiation is a pervasive communication process that is most-widely used to plan for the future, allocate resources, resolve conflicts of interests, and solve complex problems *via* mutually satisfying agreements (Jang et al., 2018).

“[Negotiations] can dramatically reshape the social and physical environments we occupy” (Jang et al., 2018, p. 318). The transformative potential of collaborative decision-making processes to lead to new practices (Asara et al., 2015) has long been recognized by scholars of social conflict. Indeed, Pruitt and Carnevale (1993, p. 15) concluded that “…[negotiation] presides over much of the change that occurs in human society. Conflict often results from dissatisfaction with the status quo, and it often leads to negotiation about how to do things differently. […] [S]ociety usually prospers if negotiation goes well and the agreements reached are mutually satisfying to the parties. Conversely, society is often harmed when negotiation goes poorly and fails to produce a mutually satisfying outcome.”

Negotiation processes can trigger change at different societal levels (the Multi-level perspective; Geels and Schot, 2007; Geels, 2011). At the micro-level (i.e., niches), at which individual actors operate, negotiation processes can promote sustainability transitions. At the meso-level (i.e., regimes), diverse stakeholders and representatives of social groups (e.g., communities, firms, private and public organizations, political parties, governmental institutions) incrementally transform the current state of society *via* negotiations (Geels, 2020). Across both levels, negotiation processes constitute an essential element of collective sense-making processes and can foster societal change (Geels, 2020).

It is important to note that “the structure and processes of negotiation are fundamentally the same at the personal level as they are at the diplomatic and corporate level” (Lewicki and Litterer, 1985). Indeed, negotiations are interactive human decision-making processes. In line with this reasoning, our conceptual article stands in the tradition of psychological and behavioral decision-making research in assuming that negotiators depart from rationality in systematic ways (e.g., Raiffa, 1982; Neale and Bazerman, 1985; Trötschel et al., 2015). In the transformation toward sustainability, negotiators are confronted with so-called “wicked problems,” which are characterized by systemic complexities, including the involvement of multiple, interdependent actors (Rittel and Webber, 1973). Beyond these social interdependencies, negotiators are also confronted with the critical element of time and temporal interdependencies, as has been emphasized in the extended conceptualization of “super wicked problems” (Levin et al., 2012; Peters, 2017).

Previous research has revealed that negotiations on sustainability issues are often ineffective and end in suboptimal solutions (Van der Gaast, 2015; Weber and Johnson, 2016; Dannenberg and Barrett, 2018) and that the involved parties, external stakeholders, and – most often – societies would benefit from more-mutually beneficial solutions (Bazerman et al., 1999). We argue that negotiation aimed at the transformation toward sustainability faces fundamental psychological barriers grounded in the conglomeration of social and temporal interdependencies. Given these conflicting interests both between people and over time, exactly how such transformation can be promoted remains unclear. In the psychological literature, two major lines of research have contributed significantly to our understanding of complex decision-making processes: first, the negotiation-research perspective (i.e., how parties resolve conflicts of interests between decision-makers), and second, the individual decision-making perspective (i.e., how decision-makers resolve conflicts between present- and future interests). These two research perspectives have been the focus of a long-standing dialog that has spurred innovation across and beyond lines of research (Raiffa, 1982; for a review, see Tsay and Bazerman, 2009). In the present contribution, we seek to reinvigorate this traditional dialog between the two psychological research areas and address key barriers and drivers in the transformation toward sustainability.

Given that the transformation toward sustainability faces super wicked problems (Levin et al., 2012), including conflicts between people and over time, these conflicts should be considered jointly rather than in isolation. We posit the existence of an interplay between inter- and intrapersonal conflicts (see Thompson and Gonzalez, 1997). Politicians, for instance, “[must] navigate political conflict over climate policy in Congress […] and within themselves” (Van Boven et al., 2018). Importantly, we believe that the web of interplay between conflicts is difficult to disentangle because negotiators must simultaneously integrate their own interests with those of their counterparts and reconcile their present- and future interests. The interplay between conflicts therefore acts as a significant barrier to the transformation toward sustainability (e.g., Weber and Johnson, 2016). To explicitly delineate the concrete challenges that arise from this interplay between inter- and intrapersonal conflicts, we introduce the concept of interdependent conflicts. We propose that a solution to one conflict (e.g., between decision-makers) impacts the solution to concurrent conflicts (e.g., within decision-makers). Consequently, interdependent conflicts can only be resolved efficiently by considering them simultaneously (see super wicked problems, Levin et al., 2012).

By developing a framework of interdependent conflicts, we contribute to existing research on decision-making and negotiation in the transformation toward sustainability in various ways. First, we provide a unifying structure for complex and interdependent decision-making processes. Second, taking the negotiation perspective, we seek to expand existing research by introducing a temporal dimension (i.e., negotiation agreements with short-term and long-term consequences). Third, from a multi-level perspective, we offer a systematic link between psychological negotiation research and transition management and highlight negotiation processes at different societal levels. Fourth, from an applied perspective, we aim to provide a more-comprehensive understanding of psychological conflicts in the transformation toward sustainability and to offer potential leverage points with hands-on tools for interventions that foster sustainable solutions. In essence, we seek to encourage future research to further examine human decision-making processes in the context of interdependent conflicts with the goal of fostering the transformation toward sustainability.

## The Framework of Interdependent Conflicts

**Figure 1:**
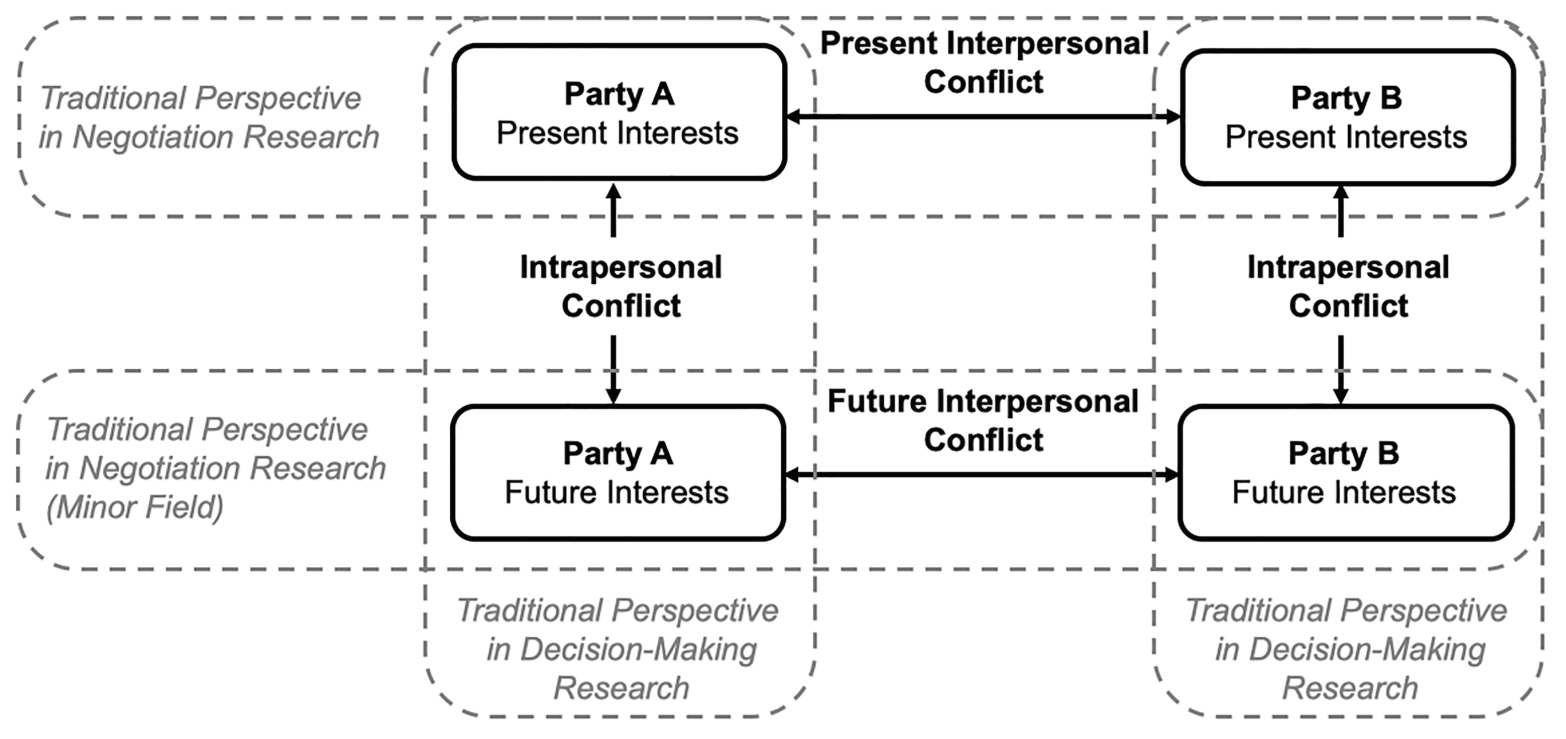
The framework of interdependent conflicts at the individual level. Figure shows the integration of traditional research fields (vertical and horizontal gray-framed areas) into our unifying framework of interdependent conflicts. The framework distinguishes between present interpersonal conflict, intrapersonal conflict emerging for each party, and future interpersonal conflict. These conflicts naturally co-occur and interdependently affect one another.

Based on the assumption that conflict resolutions depend on one another in the social and temporal dimensions, we derive a basic structure for the framework by distinguishing between three psychological conflicts. The involved parties may experience (1) *present interpersonal conflict* between their own and their counterparts’ present interests. This type of conflict has traditionally been investigated by social-conflict- and negotiation research (e.g., De Dreu et al., 2000). Simultaneously, each party may experience (2) *intrapersonal conflict* between their present- and future interests (i.e., the conflict emerges for each party individually). This type of conflict has predominantly been studied by individual decision-making research (e.g., Frederick et al., 2002). Finally, the two parties may also experience (3) *future interpersonal conflict* between their own and their counterparts’ future interests. Very few studies have investigated outcome delays and the efficiency of negotiated agreements found in this type of conflict (e.g., Okhuysen et al., 2003; Henderson et al., 2006). The parsimonious framework focuses explicitly on dyadic, two-party conflicts of interests and on two instances over time (i.e., present- and future interests).^1^ Figure 1 illustrates the proposed framework of interdependent conflicts for individual decision-makers.

Our paper is structured as follows: To establish our framework, we first introduce interdependent conflicts at the individual level. In so doing, we review the existing literature, outline characteristic psychological processes, derive propositions, and conclude with an intervention approach to addressing the proposed problems at the individual level. Second, we scale up our framework from the individual-group to the social-group level to establish interdependent conflicts as an interplay between inter- and intragenerational conflicts. We then follow the same structure as at the individual level.

## Introducing Interdependent Conflicts at the Individual Level

### Interpersonal Conflicts

Interpersonal conflicts emerge whenever two or more parties perceive their views or interests as being incompatible (Jehn, 1995), and negotiation is the decision-making process that parties with divergent interests use to reconcile their differences (Pruitt and Carnevale, 1993). Traditionally, the interest structure of interpersonal conflicts has been a central element of theorization and research (e.g., Gelfand et al., 2011). In general, the literature distinguishes between convergent- and divergent-interest structures: (1) When parties have convergent interests, these interests are compatible, and no interpersonal conflict emerges. By contrast, when parties have divergent interests, these interests can be (2a) diametrically opposed, resulting in a distributive-interest structure (i.e., a zero-sum structure without opportunities for exploring integrative, win-win solutions). In zero-sum negotiations, the best solution for both parties is a compromise (Pruitt and Carnevale, 1993). When parties have divergent interests, these interests can also be (2b) opposed, but since the parties have different priorities, they form an integrative-interest structure, which includes mutually beneficial trade-off opportunities and allows the parties to explore integrative agreements (i.e., win-win agreements; Pruitt and Carnevale, 1993). In contrast to compromise agreements, integrative agreements create value for both parties and therefore leave them better off than would a compromise (e.g., Bazerman et al., 1985). Importantly, in order to exploit integrative potential and reach mutually beneficial, transformative solutions, parties must consider their own and their counterparts’ underlying interests and coordinate them *via* negotiations.

In interpersonal conflicts, negotiators typically display the detrimental psychological tendency to devalue their counterparts’ interests (Thompson and Hastie, 1990; Babcock and Loewenstein, 1997; Curhan et al., 2004). Parties therefore have a biased idea of how to resolve a social conflict in favor of their own interests. Pinkley et al. (1995) demonstrated that negotiators devalue their counterparts’ interests and thus create suboptimal agreements even though the parties have complete information on their counterparts’ interests. As parties have a basic propensity toward interpersonal devaluation, resolving interpersonal conflicts is difficult and often leads to suboptimal agreements (Schelling, 1958; Bazerman and Neale, 1992).

### Intrapersonal Conflicts Over Time

#### The Individual Decision-Making Perspective

Decision-makers who experience intrapersonal conflict must make a choice between different alternatives that entail consequences that occur at different times (e.g., Soman et al., 2005). People must weigh immediate against future utility (Loewenstein, 1988) and thus make “trade-offs among costs and benefits occurring at different times” (Frederick et al., 2002). In the transformation toward sustainability, intrapersonal conflicts are ubiquitous and challenging to decision-makers, for instance, when choosing between maintaining the status quo or developing an alternative with substantial long-term benefits (Weber, 2017).

Research has demonstrated that people tend to temporally devalue their own future interests relative to their immediate ones (for a review, see Frederick et al., 2002). As individuals put a premium on immediate benefits, they often prefer smaller, immediate benefits over larger, later ones (Weber, 2017). Hardisty and Weber (2009, p. 329) describe this human tendency as a “strong desire, all things being equal, to get things now.” Decision-makers therefore have a biased idea in favor of their present interests in terms of how to resolve the temporal conflict.

#### The Negotiation Perspective

Social-conflict research metaphorically describes intrapersonal conflicts as two psychological states with opposing interests in which one party seeks to protect present interests and the other to protect future interests (Bazerman et al., 1998). Schelling (1984, p. 58) describes this situation with the following metaphor: “Everybody behaves like two people, one who wants clear lungs and long life and the other who adores tobacco, or one who wants a lean body and the other who wants dessert… the ‘straight’ one often in command… but the wayward one needing only to get occasional control to spoil the other’s best-laid plans.”

Read et al. (1999) indicate that such intrapersonal conflict can have similar interest structures to interpersonal conflict. (1) When a decision-maker has convergent interests, present- and future interests are compatible, and no intrapersonal conflict emerges. When one decision-maker has divergent interests, present- and future interests can be (2a) diametrically opposed, resulting in a distributive-interest structure over time. In this case, the decision-maker prefers the diametrically opposed option now as opposed to later. Alternatively, the decision-maker’s present- and future interests can also be (2b) opposed but have different priorities, resulting in an integrative-interest structure over time. Preference-consistent trade-offs can therefore also reconcile a party’s interests over time in individual decision-making. Read et al. (1999, p. 184) suggest that “analogously [to interpersonal conflicts], individual decision-makers can reach integrative agreements with themselves, if they consider the possibility of trade-offs across the many choices that they face.” To reach efficient solutions in an intrapersonal conflict, decision-makers must consider their own present- and future interests and reconcile them by negotiating with themselves over time (Bazerman et al., 1998). Therefore, researchers argue that intrapersonal conflicts are as difficult to resolve as interpersonal conflicts (Bazerman et al., 1998).

### Characteristic Psychological Processes in Inter- and Intrapersonal Conflicts

In the following sections, we highlight the central psychological processes involved in the interplay between interdependent conflicts based on the reviewed literature. We remain fully aware that other cognitive, motivational, and affective processes may also contribute to inefficient conflict resolution.

#### Interpersonal and Intertemporal Devaluation

As parties are prone to devalue others’ present interests and their own future interests (Babcock and Loewenstein, 1997; Frederick et al., 2002), we conclude that devaluing interests is likely the dominant psychological tendency in interdependent conflicts. Decision-makers face three distinct interests in addition to their own present interests: their counterparts’ present interests, their own future interests, and their counterparts’ future interests. Solutions to interdependent conflicts are hence impaired by either interpersonal devaluation, intertemporal devaluation, or both: In a present interpersonal conflict, a party socially devalues their counterparts’ present interests. In an intrapersonal conflict, a party temporally devalues their own future interests. In a future interpersonal conflict, a party interpersonally and intertemporally devalues their counterparts’ future interests. In line with previous research (Wade-Benzoni and Tost, 2009; Charlton et al., 2013), devaluation should be strongest in future interpersonal conflicts due to the duality of interpersonal and intertemporal devaluation.

#### Outcome Interdependence and Decisional Control

In addition to the processes of interpersonal and intertemporal devaluation, outcome interdependence and decisional control play an important role in interdependent conflicts. Following Interdependence Theory (Kelley and Thibaut, 1978), the structure of any given interdependence situation can be described in terms of specific features that aid in the understanding of psychological processes (Rusbult and Van Lange, 1996). Outcome interdependence and decisional control differ systematically across types of psychological conflicts. Specifically, the degree of outcome interdependence varies across inter- and intrapersonal conflicts. Whereas Party A’s outcomes are interdependent on Party B’s outcomes (interpersonal conflict), Party A’s future outcomes are purely dependent on its present outcomes (intrapersonal conflict). Consequently, parties’ decisional control also ranges across conflicts, from joint control in interpersonal conflicts to actor control in intrapersonal conflicts.^2^

In intrapersonal conflict, decision-makers face a situation with outcome dependence and full actor control and can decide how to resolve a conflict between their own present- and future interests independently of their counterparts. Herrnstein and Prelec (1991) describe actor control with a metaphor from the courtroom: The moment that a temporal decision is made, the actor functions as both “judge and jury.” In intrapersonal conflicts, parties have full actor control to simply overrule their own future interests and only serve their present interests, or vice-versa (see also Loewenstein, 1996).

By contrast, in interpersonal conflicts, parties face a situation with outcome interdependence and joint control – that is, both parties’ outcomes are mutually dependent on the decisions and actions of their counterparts. Parties thus have joint control and must therefore coordinate their decisions with those of their counterparts. Joint control has been metaphorically described by conflict scholars as the “negotiation dance” (Raiffa, 1982) to highlight the coordination of decisions and actions in interpersonal conflicts.

Based on the distinction between full actor and joint control, parties could perceive of having different degrees of freedom in resolving their conflicts of interests over time and between people. Specifically, conflicts over time (i.e., outcome dependence) may be resolvable *via* actor control. By contrast, conflicts between people (i.e., outcome interdependence) may only be resolvable *via* joint control. Due to these differences across conflicts, parties may experience more constraints in resolving conflicts of interests with their counterparts (i.e., joint control) compared with resolving conflicts of interests with themselves (i.e., actor control). We therefore conclude that negotiators tend to prioritize the resolution of inter- over intrapersonal conflicts because solutions between people require interpersonal coordination, whereas solutions over time are less constrained by coordination with other parties.

### Parties’ Consideration of Interdependent Conflicts

Building on the above-mentioned research, our framework of interdependent conflicts postulates how parties cognitively process the interplay between different psychological conflicts. In contrast to a rational approach in which parties cognitively process interdependent conflicts in a comprehensive, unbiased way (i.e., by considering all the consequences of their actions equally), we hypothesize that parties systematically prioritize the consideration of certain conflicts in a biased way.

#### Prioritizing the Consideration of Interdependent Conflicts

*Proposition 1: In interdependent conflicts, parties prioritize the consideration of present interpersonal conflicts (first priority) over intrapersonal conflicts (second priority) and future interpersonal conflicts (third priority)*.

**Figure 2:**
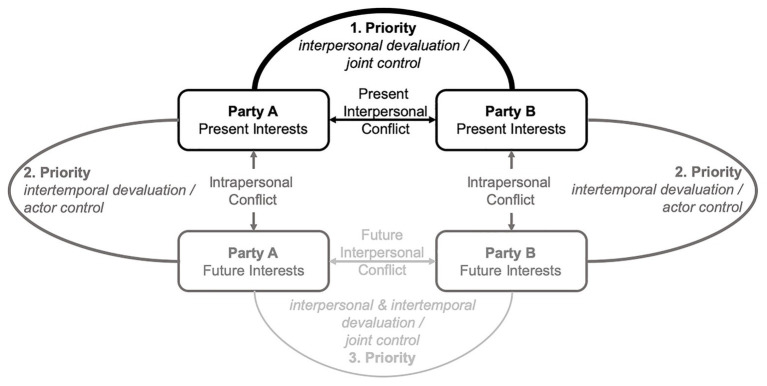
Prioritized consideration of interdependent conflicts. We propose that parties prioritize present interpersonal conflicts (first priority) over intrapersonal conflicts (second priority) and future interpersonal conflicts (third priority).

These priorities are derived both from parties’ tendency to discount their future interests (Frederick et al., 2002) and to devaluate their counterparts’ interests (Babcock and Loewenstein, 1997) as well as from the parties’ differences in decisional control (Kelley and Thibaut, 1978). When considering present interpersonal conflicts, parties devalue their counterparts’ present interests only on the interpersonal dimension. When considering intrapersonal conflicts, parties devalue their future interests only on the temporal dimension. However, when considering future interpersonal conflicts, they devalue not only their own future interests on the temporal dimension but also their counterparts’ future interest on the interpersonal and intertemporal dimension. This devaluation should lead to a more-pronounced consideration of the present inter- and intrapersonal conflict compared with future interpersonal conflicts. However, as detailed above, in addition to devaluation, parties also experience less decisional control and more constraints when resolving inter- over intrapersonal conflicts. Together, this observation should lead to a prioritized consideration of present interpersonal conflicts (first priority) over intrapersonal conflicts (second priority) and future interpersonal conflicts (third priority; see Figure 2). Consequently, parties’ prioritization of interdependent conflicts should impair a balanced and comprehensive consideration of conflicts. Noteworthy, such a prioritization of conflicts should result in an unbalanced and biased way of processing interdependent conflicts.

In line with this reasoning, prioritizing the consideration of conflicts should also determine which conflict is resolved at the cost of another.^3^ We postulate that conflicts with a higher-order priority (e.g., a present interpersonal conflict) are likely to be resolved at the cost of resolving conflicts with a lower-order priority (e.g., an intrapersonal conflict). This biased prioritization may have important implications for resolving interdependent conflicts and threaten the transformation toward sustainability.

Initial support for our assumptions can be found in a survey study (Drory and Ritov, 1997) that investigated conflict-management strategies when parties experienced only an interpersonal conflict vs. both an interpersonal conflict and an intrapersonal conflict. Parties preferred more-cooperative strategies for resolving the present interpersonal conflict when they experienced the intrapersonal conflict simultaneously as compared with when they did not. Similarly, parties that experienced interdependent conflicts were more inclined to collaborate with their counterparts when the intrapersonal conflict between present interests and long-term adverse consequences was made explicit (vs. implicit; Ritov and Drory, 1996). This finding is in line with recent research revealing that parties value agreements over impasses when dealing with present interpersonal conflicts, even if the impasse would lead to more-profitable outcomes than would the achieved agreement (Tuncel et al., 2016).

#### Effects of Priorities in the Consideration of Conflicts on the Quality of Agreements

*Proposition 2: Prioritizing the consideration of conflicts determines the extent to which parties can exploit integrative potential and reach integrative agreements*.

To resolve interdependent conflicts in an integrative way, decision-makers must consider their interests in a comprehensive rather than in an isolated, prioritized way. From a rational perspective, parties can maximize the utility of a solution (Raiffa, 1982) by making integrative trade-offs between their own and their counterparts’ interests (i.e., interpersonal conflict) and between their present- and future interests (i.e., intrapersonal conflict). Such trade-off opportunities can only be exploited when parties consider the conflicts in a comprehensive, unbiased way. However, the predicted tendency to prioritize conflicts should lead to a biased, prioritized consideration and therefore hinder parties in exploiting integrative potential. Specifically, if integrative potential is found in the intrapersonal conflict or even in the future interpersonal conflict, parties should neglect these trade-off opportunities and instead seek to resolve the present interpersonal conflict. Consequently, prioritizing conflict consideration can be particularly detrimental because parties do not consider all trade-off opportunities in a comprehensive, unbiased way and may thus overlook mutually beneficial and transformative solutions.

O’Connor et al. (2002) showed that responders in a simulated-ultimatum game rejected more bids (i.e., forewent favorable solutions in an intrapersonal conflict) when instructed to focus on the present interpersonal conflict compared with the intrapersonal conflict. This finding provides initial support for our assumptions on the detrimental effects of prioritizing interdependent conflicts.

### An Intervention Approach to Addressing a Prioritized Consideration of Conflicts

We assume that prioritizing the present interpersonal conflict is caused – in part – by constraints in decisional control. Resolving interpersonal conflicts requires negotiating between parties to overcome divergent interests, whereas resolving intrapersonal conflict does not require negotiating to overcome divergent interests in the present or future. To balance the consideration of interdependent conflicts, we propose also applying a negotiation strategy to intrapersonal conflicts over time (Bazerman et al., 1998). Negotiating “with oneself” should help parties reach integrative solutions over time and raise the priority of intrapersonal conflicts.

**Figure 3:**
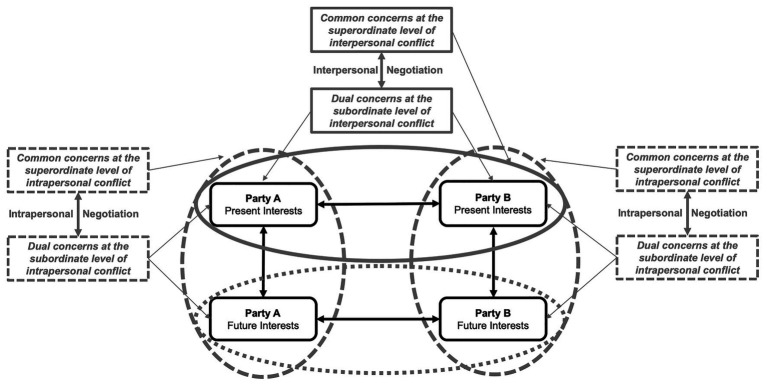
The negotiation-with-oneself strategy for balancing the consideration of interdependent conflicts at the individual level. The horizontal ellipses show how common concern can lead to integrative negotiation processes between parties. The vertical ellipses show how common concern can lead to integrative negotiation processes over time.

Social-conflict research has revealed that integrative solutions are particularly likely when each negotiator (1) has a strong concern for his or her own outcomes (dual concerns at a subordinate level; Pruitt and Carnevale, 1993; De Dreu et al., 2000) and (2) takes both parties’ common interests into consideration (common concerns at a superordinate level; Rhoades and Carnevale, 1999; De Dreu et al., 2000; Trötschel et al., 2011, 2021). Accordingly, parties should be concerned about (1) their present- and future interests (dual concerns at a subordinate level) and (2) their common interests over time (common concerns at a superordinate level). Considering dual and common concerns over time should trigger negotiating with oneself, and this strategy should raise the intrapersonal conflict to the same level of priority as the interpersonal conflict. Simultaneously, raising the priority of intrapersonal conflicts by negotiating with oneself should also lead to an increase in the priority of future interpersonal conflicts. Overall, we posit that combining interpersonal and intrapersonal negotiation should lead to a balanced, unbiased, comprehensive consideration of interdependent conflicts (see Figure 3).

#### Applying the Intervention Approach to the Transformation Toward Sustainability

Negotiations play a vital role in community-led grassroots innovations that are niche spaces supporting local-scale transitions toward sustainability (e.g., Raven et al., 2008; Seyfang and Haxletine, 2012; Ornetzeder and Rohracher, 2013). Grassroots initiatives have been shown to foster change in diverse areas, such as mobility or energy (Ornetzeder and Rohracher, 2013). However, a crucial success factor for exploiting the transformative potential of grassroots innovations is the successful negotiation and mutually-beneficial conflict resolution. Conflicts emerge because local partners and stakeholders of such an initiative may have at least some common interests but may also have opposing interests in reaching their shared objectives. For instance, individual owners of cooperative housing apartments may share their interest in investing in energy-efficient buildings, but may have diverging interests in the potential pathways to reach this energy transition. Some of the owners may prefer to install solar panels on the rooftop, whereas others may prefer to maintain the rooftop accessible for the residents and to use other energy sources for powering the building energy-efficiently. As they can only reach their objectives jointly, the cooperative owners must negotiate strategies that lead to the intended transformation of existing structures. However, all involved actors may enter negotiations by positioning their interests in their immediate and local context that may hinder the implementation of the pathway toward innovation. Both our framework of interdependent conflicts and the suggested intervention approach of intrapersonal negotiations for reconciling one’s present- and future interests may help to facilitate successful negotiations in grassroots innovations. Therefore, implementing the proposed intervention approach in the context of community-led grassroots initiatives requires that individual actors are concerned with their dual interests in the present and future at a subordinate level as well as with their common interests at a superordinate level. At a subordinate level, future interests come into play when the involved actors formulate long-term goals, develop a vision, and specify their expectations for the transition toward sustainability. Present interests may guide decision-making when searching for pathways to implement the innovation. Additionally, at the superordinate level, actors should share the common concern that radical innovation will lead to the intended transformation toward sustainability. When actors consider their dual and common concerns, intrapersonal negotiation may be initiated, and a prioritized consideration of conflicts may be debiased. As a consequence, negotiation processes between local actors may be improved and lead to more-mutually beneficial and transformative solutions for the societal transformation sparked by grassroots initiatives.

#### Tools for Implementing the Intervention Approach

Tools for implementing the negotiation-with-oneself strategy can be derived from both decision-making- and social-conflict research. Decision-making research suggests that an increasing similarity between one’s present- and future self may trigger a party’s readiness to negotiate with themself (e.g., Bartels and Urminsky, 2011; Hershfield, 2011; Urminsky, 2017). Alternatively, changing the primary default consideration from present- to future interests may also stimulate intrapersonal negotiations (Weber et al., 2007; Sunstein and Reisch, 2013). Social-conflict research suggests that perspective-taking of one’s own future interests may also help induce negotiations with oneself over time and balance the consideration of interdependent conflicts (Galinsky et al., 2008; Trötschel et al., 2011). Furthermore, learning approaches that support analogous reasoning in transferring integrative insights from one type of psychological conflict to another could facilitate interdependent-conflict resolution (Thompson and DeHarpport, 1994; Gillespie et al., 1999; Nadler et al., 2003; Kim et al., 2020).

Although interventions may support negotiators in reaching mutually beneficial, transformative solutions, reaching integrative solutions at the level of social groups has been shown to be even more challenging (Loschelder and Trötschel, 2010; Trötschel et al., 2010). However, the transformation toward sustainability most-often requires negotiations between social groups, such as between larger institutions or organizations that represent certain interests (Majer et al., 2018). Compared with interpersonal conflict, intergroup conflict stands out in terms of the distinct psychological processes involved. To further elucidate the psychological barriers to and drivers of interdependent conflicts at the group level, we next scale our framework up and focus on intergenerational conflict. Such situations include central psychological barriers that hinder us from taking dramatic action in the transformation toward sustainability (Ehrlich and Ehrlich, 2013).

## Introducing Interdependent Conflicts at the Level of Social Groups: The Interplay Between Intra- and Intergenerational Conflicts

At the zenith of the COVID-19 pandemic in July 2020, the European Union agreed on the largest budget and financial package in its history to address the aftermath of the once-in-a-century-pandemic crisis. This negotiation had implications not only for member states within the present generation but also for their successor generations to come. The talks lasted almost 100 h because the member states’ contributions were heavily disputed. After an agreement had been reached, Chancellor Merkel was relieved that Europe had shown that it can come together after all (Erlanger and Stevis-Gridneff, 2020). However, other European politicians criticized the fact that the funds for important future EU projects had been cut back to reach a deal between the member states (DLF, 2020).

**Figure 4:**
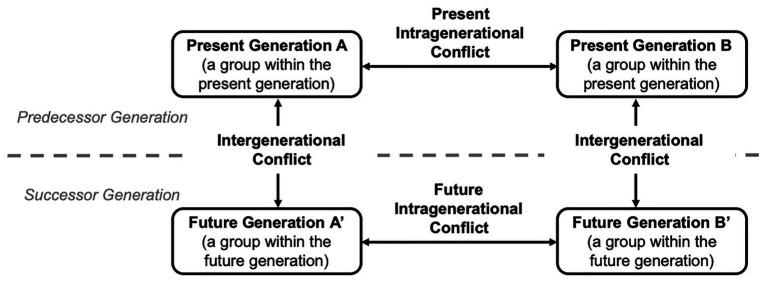
The framework of interdependent conflicts in the intergenerational context. Figure 4 displays the interplay between the arising present intragenerational conflict (i.e., the conflict between different groups within the present generation), the intergenerational conflict (i.e., the conflict between the predecessor and successor generation of a group), and the future intragenerational conflict (i.e., the conflict between different groups within the future generation).

This example can be systematically structured using the framework of interdependent conflicts. Conflicts in the transformation toward sustainability include a social dimension between groups (i.e., intragenerational conflict between different groups within a current generation) and a temporal dimension between generations over time (i.e., intergenerational conflict between the predecessor and successor generation of a single group; Sherstyuk et al., 2016; Bosetti et al., 2020). In line with our framework, scholars have proposed that “many real-world intergenerational dilemmas [i.e., over time] are confounded by intragenerational social dilemmas [i.e., between groups]” (Wade-Benzoni et al., 2008). Following this reasoning, we systematically differentiate between three types of psychological conflicts (Figure 4): (1) *present intragenerational conflict* (i.e., between different groups within the present generation); (2) *intergenerational conflict* (i.e., between the predeceasing present generation and succeeding future generation of a single group); and finally, (3) *future intragenerational conflict* (i.e., between different groups within the future generation; see Footnote 1).^4^

### Intergroup Conflicts (i.e., Intragenerational Conflict)

A group consists of two or more individuals connected by social relationships (Forsyth, 2014). These relationships can be established objectively *via* outcome interdependence between individuals, which induces the formation of groups (Lewin, 1948). Alternatively, relationships can also be established subjectively by assigning memberships to in-groups or out-groups to oneself and others based on similarity (Tajfel, 1981). The conflicts between groups can be described as incompatibilities in the different groups’ values and/or goals, which may be caused by outcome interdependence and/or perceived similarity (Boehm et al., 2020). This idea implies that intergroup conflict may involve not only economic interests but also categorization as an in- or out-group. In the transformation toward sustainability, the two foundations of intergroup conflict often arise in combination (e.g., Majer et al., 2018; Schuster et al., 2020).

Early theorizations on the causes of intergroup conflict focused on economic interests in (scarce) resources as the root of competition in intergroup conflict (Sherif and Sherif, 1953; Sherif, 1961; Campbell, 1965). When comparing interpersonal and intergroup interactions, research found that intergroup relations are more competitive than are interpersonal relations (Wildschut and Insko, 2007) and suggested that fear and greed explain this discontinuity effect in intergroup interactions (Wildschut and Insko, 2007). Specifically, fear is based on the expectation that the other group will maximize its outcome, which poses a threat to the given group and increases competition. By contrast, greed is based on the expectation that the other group will tend to cooperate, which makes the other group vulnerable to the given group’s greed and increases competition.^5^

However, another line of research suggests that merely categorizing oneself and others as members of an in- and out-group, respectively, is sufficient to induce intergroup conflict (Tajfel and Turner, 1979, 1986). Specifically, Self-Categorization Theory posits that individuals are motivated to make themselves positively distinct from others by comparing themselves to others on relevant dimensions (Turner et al., 1987). If comparisons are favorable for the in-group relative to the out-group, people can make themselves positively distinct, with beneficial and direct consequences for their self-concept and self-esteem. Evidence shows that people strive for positive distinctiveness (for an overview, see Boehm et al., 2020), which can be obtained *via* different strategies, including social competition, for instance, by discriminating the out-group.

Overall, greed and fear as well as the need for positive distinctiveness all contribute to intergroup devaluation. Greed and fear are particularly pronounced when outcome interdependence exists. However, the need for positive distinctiveness can be explained by the psychological process of self-categorization as an in- or out-group member.

### Intragroup Conflicts Over Time (i.e., Intergenerational Conflict)

In contrast to intergroup conflicts within a generation (e.g., Barrett and Dannenberg, 2012), much-less work has focused on intergenerational conflicts over time (e.g., Hauser et al., 2014). From a psychological perspective, intergenerational conflicts (Wade-Benzoni and Tost, 2009) are characterized as decisions in which the interests of present decision-makers stand in conflict with those of future others. Such intergenerational conflicts have distinctive features as compared with intergroup (i.e., intragenerational) conflicts (Wade-Benzoni and Tost, 2009).

Specifically, outcomes are not reciprocally interdependent in intergenerational conflicts. Instead, the outcomes of the future generation are fully determined by the present generation. Present generations therefore have complete actor control without the need to coordinate their interests with future others. Consequently, future generations have no voice in intergenerational conflicts (see outcome interdependence; Kelley and Thibaut, 1978). In addition, present generations do not have to bear the long-term consequences of their decisions and actions because they are not part of the generation that experiences the consequences. Furthermore, no direct or indirect reciprocity between the present- and future generation is possible (Wade-Benzoni and Tost, 2009). The future generation cannot give anything back or punish the present generation. This lack of direct or indirect reciprocity also implies a lack of communication between the present- and future generations. Importantly, in intergroup conflicts between different groups within a current generation, reciprocity, and communication have been shown to increase cooperation and lead to more-mutually beneficial solutions (e.g., Tavoni et al., 2011; Yoeli et al., 2013). However, as the direct experience of consequences, reciprocity, and communication are ruled out in intergenerational conflict, cooperation, and integrative solutions between the present- and future generation are further exacerbated. In intergenerational conflicts, the future generation’s outcomes depend on the present generation’s beneficence (i.e., intergenerational beneficence), which is often lacking (Sherstyuk et al., 2016; Bosetti et al., 2020). To increase intergenerational beneficence, it is therefore necessary for a perceived similarity between the present- and future generation to exist and for the present generation to identify with the future generation.

### Characteristic Psychological Processes in Intra- and Intergenerational Conflicts

#### Intergroup Devaluation

Intergroup devaluation can be explained by the processes of greed and fear in intergroup relations as well as by the need for positive distinctiveness in comparison with the out-group. Intergroup devaluation has been found to be particularly prominent in present- and future intragenerational conflicts, which renders these conflicts difficult to resolve.

#### Intergenerational Devaluation (i.e., Intergroup- and Intertemporal Devaluation)

Intergenerational conflicts are difficult to resolve because intergroup- and intertemporal devaluation jointly impede integrative conflict resolution. The future generation’s interests are devalued temporally. In addition, intergroup devaluation arises because the present- and future generations are typically not part of the same collective. Both intergroup- and intertemporal devaluation are additive components of intergenerational devaluation, which is the major barrier to integrative solutions in intergenerational conflicts (Wade-Benzoni and Tost, 2009). Although the degree of intergenerational devaluation should depend on perceived similarities between the present- and future generation, in general, the need for positive distinctiveness should be more-pronounced in intragenerational conflicts between distinct groups within the present generation. However, in the case of intergenerational conflicts, intergroup- and intergenerational devaluation can accumulate and lead to severe devaluation against the opposing groups’ successor generation in the future.

#### Outcome Interdependence and Decisional Control

Outcome interdependence in intragenerational conflict only exists between the two different groups within the present generation. In intergenerational conflict over time, however, future generations outcomes fully depend on the present generation. Concerning decisional control (Kelley and Thibaut, 1978), intragenerational conflict can only be resolved *via* joint control because one group within the present generation must coordinate its interests with another group of the same generation. By contrast, the present generation has full actor control in intergenerational conflicts because this generation fully determines the consequences for the succeeding future generations.

### Parties’ Consideration of Interdependent Conflicts Across Generations

In line with the general assumption of our framework of interdependent conflicts, we postulate that social groups cognitively process different psychological conflicts in a biased way. This idea stands in contrast to a rational approach in which groups cognitively process interdependent conflicts in a comprehensive, unbiased way (i.e., they equally consider all consequences of their actions).

#### Prioritizing Interdependent Conflicts Within and Between Generations

*Proposition 3: In interdependent conflicts at the social-group level (i.e., generations), parties prioritize the consideration of present intragenerational conflicts (first priority) over intergenerational conflicts (second priority) and future intragenerational conflicts (third priority)*.

Social groups have a tendency to prioritize present intragenerational conflicts because joint control with the other group within the present generation places constraints on the decision-making process and requires coordination between groups. This joint control stands in contrast to intergenerational conflicts over time, which should be given second priority because the present generation has full actor control when it comes to resolving these conflicts. In line with this reasoning, future intragenerational conflicts should be given third priority because in addition to intergenerational devaluation, the need for positive distinctiveness from the other group (i.e., intergroup devaluation) also contributes to the prioritization of these conflicts.

These priorities also determine which conflict will be resolved at the cost of another. Conflicts of higher priority may be resolved at the cost of lower-priority conflicts because present intragenerational conflicts should receive more consideration than intergenerational conflicts or future intragenerational conflicts. Prioritizing the consideration of interdependent conflicts thus has important implications for the transformation toward sustainability.

Recent research has found initial support for Proposition 3 (Sherstyuk et al., 2016) by showing that adding the dimension of intergenerational conflict over time to the dimension of intragenerational conflict renders conflict resolution between parties more short-sighted.

#### Effects of Priorities in the Consideration of Conflicts on the Quality of Agreements

*Proposition 4: A prioritized consideration of conflicts determines the extent to which social groups (i.e., generations) can exploit the integrative potential and reach integrative agreements*.

To achieve mutually beneficial, transformative solutions at the group level, a balanced and unbiased consideration of all conflicts (rather than a prioritized consideration) is necessary. However, we assume that the involved groups prioritize conflicts with detrimental consequences. Specifically, parties consider the coordination of diverging interests in higher-priority conflicts to a greater extent than in lower-priority conflicts. Integrative potential and the trade-off opportunities embedded within lower-priority conflicts are therefore less-likely to be discovered. A prioritized, biased consideration of conflicts should thus result in suboptimal solutions for involved groups. In other words, resolving interdependent conflicts should be transformative and mutually beneficial if future generations’ interests are considered in an unbiased and balanced way.

Jacquet et al. (2013) provided initial evidence for Proposition 4 by experimentally demonstrating that when a temporal dimension is introduced in intergroup conflicts, conflict resolution is less optimal than when the intergroup conflict has no long-term consequences.

### An Intervention Approach to Addressing a Prioritized Consideration of Interdependent Conflicts Across Generations

Based on research on social conflict and negotiation (e.g., De Dreu et al., 2000) and on intergroup conflict (e.g., Dovidio et al., 2000), we develop an intervention approach tailored to balance the consideration of interdependent conflicts between social groups. Research has shown that the perception of belonging to distinct, opposed groups (“us” vs. “them”) can be changed *via* interventions (Dovidio et al., 2000). Specifically, by re-categorizing one’s own group and the other group into subgroups of one superordinate, common in-group identity (the new “we” – i.e., two subgroups within one group; Gaertner et al., 1993, 1994), intergroup conflict can be reduced. Importantly, managing intragenerational conflict *via* negotiations requires that (1) the two subgroups consider their common concerns by creating a new superordinate, common in-group identity and (2) that each subgroup maintain its distinct group membership and consider its dual concerns (i.e., creating a common in-group identity, while maintaining dual identities). If the groups consider their superordinate, common in-group identity and common concerns, while simultaneously considering their dual identities and dual concerns, intragenerational conflicts can be resolved in an integrative, unbiased way (Gaertner et al., 2016).

To balance the consideration of interdependent conflicts across social groups and time, we transfer the intervention approach from intra- to intergenerational conflict. We find the classic, common in-group-identity approach particularly suitable for stimulating negotiations with future others in an integrative way. As a prerequisite, the present generation should (1) create a common in-group identity with their succeeding future generation that includes common concerns shared by the present- and future generations and (2) acknowledge their distinct dual identities over time – including dual concerns of the present- and future generations – in order to stimulate negotiations with future others.

**Figure 5:**
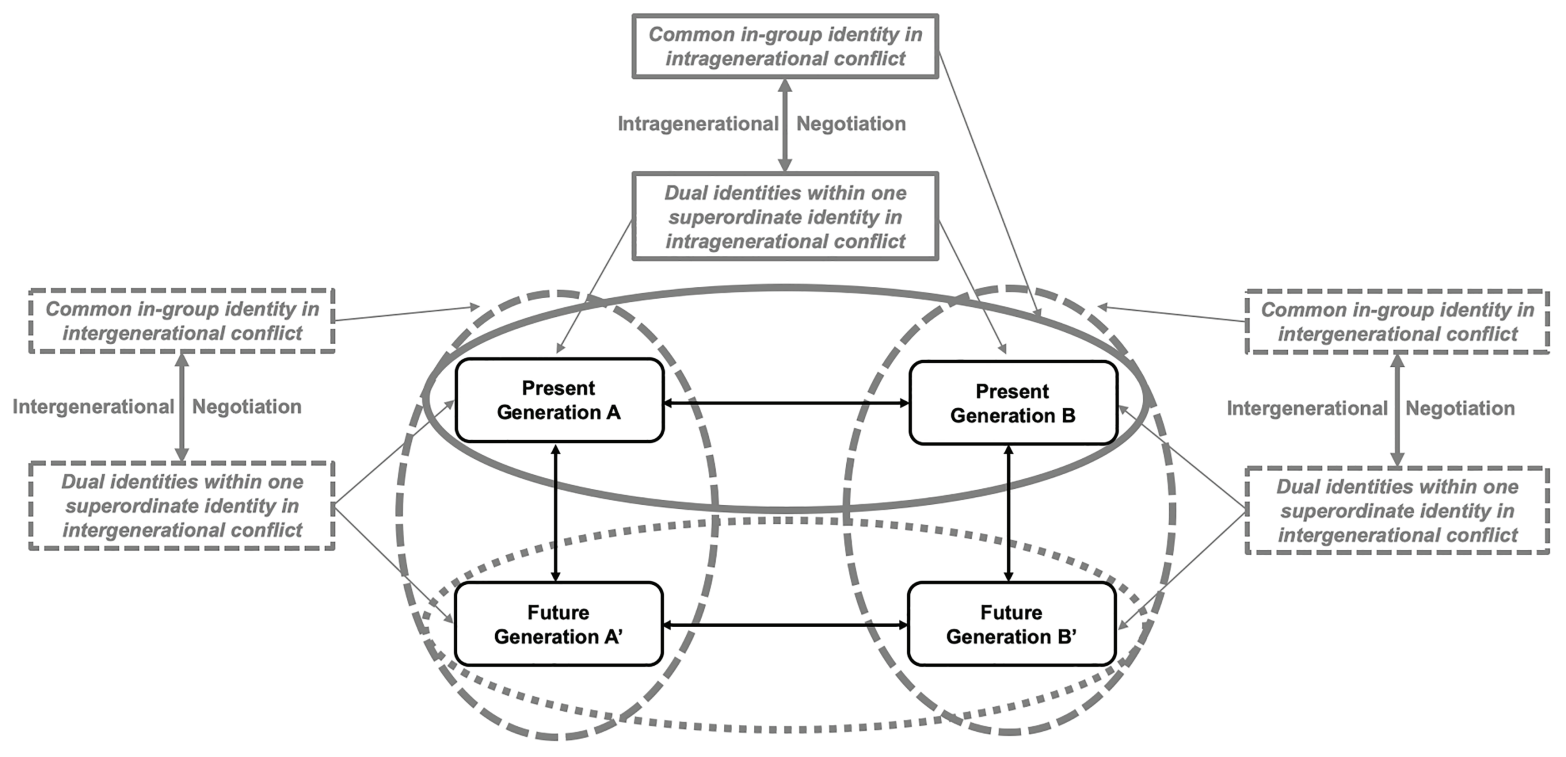
The negotiating-with-future-others strategy for balancing the consideration of interdependent conflicts at the generational level. The horizontal ellipses show how common in-group identity leads to integrative negotiation processes at the intragenerational level between groups. The vertical ellipses show how common in-group identity leads to integrative negotiation processes at the intergenerational level over time. If both groups engage in such intergenerational negotiation processes, they should also be able to balance the consideration of future intragenerational conflicts.

However, in intergenerational conflict, future generations have no voice to stand up for their concerns. As communication between present- and future generations is ruled out, a shift toward future generations’ interests is necessary to elicit negotiations with future others. We propose that present generations be held responsible for resolving intergenerational conflicts *via* negotiations. Contemporary representatives of the future generation may take responsibility for speaking up for their generations’ interests (Kamijo et al., 2017). This negotiating-with-future-others strategy combines a common in-group-identity approach with a representation of future generations in order to foster integrative solutions. Negotiating with future others also raises the priority of the intergenerational conflict compared with that of the present intragenerational conflict, thereby leading to a more-balanced consideration of interdependent conflicts. If each present generation uses the negotiating-with-future-others strategy, a more-balanced consideration of the future intragenerational conflict should also be reached. Overall, negotiating with future others should be a particularly suitable approach to balancing the consideration of interdependent conflicts and fostering mutually beneficial and transformative solutions (Figure 5).

#### Applying the Intervention Approach to the Transformation Toward Sustainability

Negotiations are also an integral part of the transition management approach (Meadowcroft, 2009; Loorbach, 2010; Schreuer et al., 2010), which typically seeks to regulate and govern fundamental processes of societal change that may take generations to realize (Frantzeskaki et al., 2012). During this transition, the sustainability value of intergenerational justice must be protected. However, the involved societal groups of the present generation may enter negotiations by positioning their interests in their direct and immediate context, thereby leading to suboptimal solutions (Loorbach, 2010). In particular, the different interest groups within the present generation may experience short-term need for compromises, whereas succeeding future generations need long-term ambitions for radical change (Frantzeskaki et al., 2012). Traditionally, transition management distinguishes between four types of circular-governance activities to facilitate sustainability transitions: strategic, tactical, operational, and reflexive activities (Loorbach, 2010). The strategic and tactical activities in the transition-management cycle are largely interest-driven and require negotiation between representatives and delegates of larger societal interest groups, organizations, or institutions that have the capacity to contribute to the vision of the transition. Particularly during the tactical-activity phase of the transition-management cycle, the development of a concrete transition agenda requires the negotiation and coordination of interests between groups within the present generation and the alignment of these interests with those of future generations. In an exemplary innovation program on future urban mobility (e.g., urban-living labs, von Wirth et al., 2018), stakeholder groups of the present generation such as local residents, public transportation services, private mobility providers, and city authorities develop transition scenarios (Sondeijker et al., 2006), which are descriptions of desirable future states that include alternative pathways for reaching them (i.e., backcasting). However, the interests of future generations should be aligned with these transition scenarios created by the delegates of the stakeholder groups within the present generation. According to our intervention approach, present delegates should create a common in-group identity with the succeeding future generation and also consider the dual identities of the present- and the future generations when developing the scenario for the urban mobility transition. In addition, a representative of the future generation could be assigned to safeguard the future generation’s interests during the development of scenarios for the urban mobility transition. Our proposed intervention approach may be particularly suitable for generating more mutually beneficial and transformative solutions in the management of transitions when interests within and between generations must be negotiated. As a result, the negotiation-with-future-others strategy may help to overcome a biased and unbalanced consideration of interdependent conflicts between societal interest groups and their successor generations.

#### Tools for Implementing the Intervention Approach

Potential tools for creating common in-group identities include placing focus on superordinate-group memberships (e.g., nations, organizations, and communities), increasing affinity with future generations (Wade-Benzoni, 2008; Arora et al., 2016), and emphasizing factors that are shared by the groups (e.g., values, fate, and goals). Alternative tools exist that may further trigger intergenerational negotiations over time by forecasting future generations’ beneficence (Bosetti et al., 2020), priming present generations with the inevitability of their own mortality (Wade-Benzoni et al., 2012), or providing advice to future generations (Sherstyuk et al., 2016). However, these tools often neglect common in-group identities and the representation of future generations, both of which are required to elicit negotiations with future others.

## General Discussion

We developed and introduced a framework of interdependent conflicts for stimulating novel research that examines individual- and joint decision-making processes in the transformation toward sustainability. The critical relevance that negotiations entail in this transformation is undisputed (Pruitt and Carnevale, 1993; Loorbach, 2010); however, it is also unanimously accepted that “negotiation will fail to achieve fundamental change unless there is a commitment to long-term change […]” (Kemp et al., 2007, p. 316). Despite this conclusion, the existing literature on negotiations and decision-making treats sustainability challenges rather unidimensionally. While negotiation- and social-conflict research primarily focus on conflict resolution in the present (Jang et al., 2018), individual decision-making often neglects the social interdependencies against which deep structural change must be negotiated and coordinated.

Typically, decision-makers must simultaneously consider their own interests and those of other decision-makers in addition to long-term future consequences for themselves and future others. We aimed to provide a novel perspective on why agreements reached *via* negotiations are often not in favor of our own or others’ long-term interests. One of the key contributions of our novel framework is that it enables an analysis of decision-making settings in the transformation toward sustainability in a more-comprehensive, unifying, and systematic way. Moreover, our framework provides a parsimonious structure for disentangling these complex conflict situations, analyzing the arising psychological phenomena, and designing interventions that tap into the psychological barriers that impede transformative solutions. At best, agreements create integrative solutions for all parties involved – not only in the present, but also over longer timespans. Our framework offers a systematic integration of the social and temporal dimensions and thereby helps in reaching these transformative and mutually beneficial solutions.

Sustainability challenges represent the largest collective-action problem ever faced by humanity (Ostrom, 2009). Joint decision-making and negotiation, cooperation, and conflict resolution are therefore inevitable in making collective progress toward sustainable living in our societies. Taking the proposed psychological barriers into account, these negotiation processes may be biased toward solutions in the present. To overcome this crucial barrier, a better understanding of the underlying psychological processes may help in guiding negotiation processes that promote forward-looking conflict resolution. The European Union’s financial and budget deal closed by the different member states is exemplary in demonstrating interdependent conflicts. On the one hand, various member states of the European Union have repeatedly shown that they can come together to jointly resolve issues of the present generation that they could not deal with individually. On the other hand, resolving conflicts between member states within the present generation may lead to costs for member states’ very own long-term interests and for those of their succeeding future generations.

The described tensions may lead to a rather skeptical view of the transformative potential of negotiations. Indeed, the challenges for parties in creating transformative solutions are difficult. However, we hope that our framework and the proposed intervention approaches might help negotiators navigate toward more-transformative solutions across different societal levels and contexts. In grassroots initiatives, small groups of societal frontrunners may initiate negotiations over innovations and, in the management of the transition, representatives of larger societal-interest groups, institutions, or organizations may negotiate their interests in contributing to the transition pathway. Thereby, negotiations may also help to bridge structural changes across societal levels. We believe that existing and potential future tools for implementing intervention approaches should be tested, adapted, and refined depending on the interdependent-conflict situation. Nevertheless, we wish to emphasize the idea that interdependent conflicts are negotiable not only between individual actors and societal groups but also within ourselves and across generations. Making use of the transformative potential of these negotiation processes may open new transition pathways toward sustainability. We, therefore, remain optimistic that negotiations as collaborative decision-making approaches are most promising for reaching transformative solutions and are our only true alternative to collaboratively achieving long-term societal prosperity (Pruitt and Carnevale, 1993). In acknowledging this belief, the framework of interdependent conflicts may provide innovative impulses for integrating and reconciling interests within planetary boundaries.

## Data Availability Statement

The original contributions presented in the study are included in the article/supplementary material; further inquiries can be directed to the corresponding author.

## Author Contributions

JM developed the theory, created the figures, and drafted the manuscript. MB, HZ, MT, and RT contributed to theory development, structuring, and revising the manuscript. All authors contributed to the article and approved the submitted version.

### Conflict of Interest

The authors declare that the research was conducted in the absence of any commercial or financial relationships that could be construed as a potential conflict of interest.

## Footnotes

^1^Besides the specified psychological conflicts in the framework, two other psychological conflicts might emerge for each party (i.e., an interpersonal conflict over time in which one party’s present interests conflict with the counterpart’s future interests and an interpersonal conflict over time in which one party’s future interests conflict with the counterpart’s present interests). These interpersonal conflicts over time directly reflect the unique characteristic of interdependent conflicts. Since conflicts are interdependent, the specified three different types of psychological conflicts in our framework can determine the parties’ interpersonal conflicts over time. Therefore, the framework of interdependent conflicts implicitly integrates these interpersonal conflicts over time. For conciseness reasons, the presented version of the framework of interdependent conflicts offers the most parsimonious version that may be extended in future research on interdependent conflicts in the transformation toward sustainability. Our reasoning also applies to interdependent conflicts at the level of social groups that we address in the latter part of the article.

^2^Another extreme is partner control (e.g., Van Lange and Balliet, 2015), which is omitted here for reasons of simplicity.

^3^Psychological conflicts may be either independent, positively interdependent, or negatively interdependent. When conflicts are independent of one another, one conflict can be resolved without any consequences for resolving the other. In current individual- and societal challenges, conflicts are rarely independent of one another (super wicked problems; (Levin et al., 2012). By contrast, in most current social issues, interdependence between conflicts occurs: Parties’ consideration of their present interests in an interpersonal conflict usually impacts their consideration of interests in the future, and vice versa. When conflicts are positively interdependent, resolving one psychological conflict also facilitates finding a solution to the other interdependent conflict. However, positive interdependence does occur in real-world settings, albeit rarely. Most importantly, though, when psychological conflicts are negatively interdependent, parties’ efforts to resolve one conflict impede efficiently resolving the other interdependent conflict. We therefore only focus on negative interdependence between conflicts in our framework.

^4^We are aware that different constellations between present- and future generations can be conceived (e.g., generations living at the same time, group representations). However, we follow the standard definition and focus explicitly on the basic situation in which the present generation (as predecessors) has no contact with the future generation (as their successors; Wade-Benzoni and Tost, 2009; Bosetti et al., 2020).

^5^Various explanatory mechanisms are discussed in the fear- and greed perspective for situations in which groups’ outcomes are interdependent, but these mechanisms lie beyond the scope of this article.

## References

[ref1] Arora P. Logg J. Larrick R. (2016). Acting for the greater good: identification with group determines choices in sequential contribution dilemmas. J. Behav. Decis. Mak. 29, 499–510. 10.1002/bdm.1892

[ref2] Asara V. Otero I. Demaria F. Corbera E. (2015). Socially sustainable degrowth as a social–ecological transformation: repoliticizing sustainability. Sustain. Sci. 10, 375–384. 10.1007/s11625-015-0321-9

[ref3] Babcock L. Loewenstein G. (1997). Explaining bargaining impasse: the role of self-serving biases. J. Econ. Perspect. 11, 109–126. 10.1257/jep.11.1.109

[ref4] Barrett S. Dannenberg A. (2012). Climate negotiations under scientific uncertainty. Proc. Natl. Acad. Sci. U. S. A. 109, 17372–17376. 10.1073/pnas.1208417109, PMID: 23045685 PMC3491499

[ref5] Bartels D. M. Urminsky O. (2011). On intertemporal selfishness: how the perceived instability of identity underlies impatient consumption. J. Consum. Res. 38, 182–198. 10.1086/658339

[ref6] Bazerman M. H. Magliozzi T. Neale M. A. (1985). Integrative bargaining in a competitive market. Organ. Behav. Hum. Decis. Process. 35, 294–313. 10.1016/0749-5978(85)90026-3

[ref7] Bazerman M. H. Moore D. A. Gillespie J. J. (1999). The human mind as a barrier to wiser environmental agreements. Am. Behav. Sci. 42, 1277–1300. 10.1177/00027649921954868

[ref8] Bazerman M. H. Neale M. A. (1992). Negotiating rationally. New York, NY: Free Press.

[ref9] Bazerman M. H. Tenbrunsel A. E. Wade-Benzoni K. (1998). Negotiating with yourself and losing: making decisions with competing internal preferences. Acad. Manag. Rev. 23:225. 10.5465/amr.1998.533224

[ref10] Boehm R. Rusch H. Baron J. (2020). The psychology of intergroup conflict: a review of theories and measures. J. Econ. Behav. Organ. 178, 947–962. 10.1016/j.jebo.2018.01.020

[ref11] Bosetti V. Dennig F. Liu N. Tavoni M. Weber E. (2020). Forward-looking belief elicitation enhances inter-generational beneficence. SSRN Electron. J. 10.2139/ssrn.3648287

[ref12] Campbell D. T. (1965). “Ethnocentric and other altruistic motives” in Nebraska symposium on motivation. ed. Levine D. (Lincoln: University of Nebrasksa Press), 283–311.

[ref13] Charlton S. R. Yi R. Porter C. Carter A. E. Bickel W. Rachlin H. (2013). Now for me, later for us? Effects of group context on temporal discounting. J. Behav. Decis. Mak. 26, 118–127. 10.1002/bdm.766, PMID: 23641123 PMC3639500

[ref14] Curhan J. R. Neale M. A. Ross L. (2004). Dynamic valuation: preference changes in the context of face-to-face negotiation. J. Exp. Soc. Psychol. 40, 142–151. 10.1016/j.jesp.2003.12.002

[ref15] Dannenberg A. Barrett S. (2018). Cooperating to avoid catastrophe. Nat. Hum. Behav. 2, 435–437. 10.1038/s41562-018-0374-8, PMID: 31097808

[ref16] De Dreu C. K. W. Weingart L. R. Kwon S. (2000). Influence of social motives on integrative negotiation: a meta-analytic review and test of two theories. J. Pers. Soc. Psychol. 78, 889–905. 10.1037/0022-3514.78.5.889, PMID: 10821196

[ref17] DLF (2020). “Den Planeten in vernünftigem Zustand hinterlassen.” Available at: https://www.deutschlandfunk.de/philosophin-ueber-generationengerechtigkeit-den-planeten-in.694.de.html?dram:article_id=482019 (Accessed August 9, 2020).

[ref18] Dovidio J. F. Gaertner S. L. Kafati G. (2000). “Group identity and intergroup relations: the common in-group identity model” in Advances in group processes. *Vol*. 17 eds. Thye S. R. Lawler E. J. Macy M. W. Walker H. A. [Bingley, UK: Emerald (MCB UP)], 1–35.

[ref19] Dreber A. Nowak M. A. (2008). Gambling for global goods. Proc. Natl. Acad. Sci. U. S. A. 105, 2261–2262. 10.1073/pnas.0800033105, PMID: 18287079 PMC2268122

[ref20] Drory A. Ritov I. (1997). Intrapersonal conflict and choice of strategy in conflict management. Psychol. Rep. 81, 35–46. 10.2466/pr0.1997.81.1.35

[ref21] Ehrlich P. R. Ehrlich A. H. (2013). Can a collapse of global civilization be avoided? Proc. Biol. Sci. 280:20122845. 10.1098/rspb.2012.2845, PMID: 23303549 PMC3574335

[ref22] Erlanger S. Stevis-Gridneff M. (2020). Angela Merkel guides the E.U. to a deal, however imperfect. The New York Times. Available at: https://www.nytimes.com/2020/07/21/world/europe/european-union-coronavirus-aid.html (Accessed July 21, 2020).

[ref23] Forsyth D. R. (2014). “The psychology of groups” in Noba textbook series: Psychology. eds. Biswas-Diener R. Diener E. (Champaign, IL: DEF Publishers).

[ref24] Frantzeskaki N. Loorbach D. Meadowcroft J. (2012). Governing societal transitions to sustainability. Int. J. Sustain. Dev. 15, 19–36. 10.1504/IJSD.2012.044032

[ref25] Frederick S. Loewenstein G. O’Donoghue T. (2002). Time discounting and time preference: a critical review. J. Econ. Lit. 40, 351–401. 10.1257/jel.40.2.351

[ref26] Gaertner S. L. Dovidio J. F. Anastasio P. A. Bachman B. A. Rust M. C. (1993). The common ingroup identity model: recategorization and the reduction of intergroup bias. Eur. Rev. Soc. Psychol. 4, 1–26. 10.1080/14792779343000004

[ref27] Gaertner S. L. Guerra R. Rebelo M. Dovidio J. Hehman E. Deegan M. (2016). “The common ingroup identity model and the development of a functional perspective: a cross-national collaboration” in The social developmental construction of violence and intergroup conflict. eds. Vala J. Waldzus S. Calheiros M. M. (Cham, Switzerland: Springer International Publishing), 105–120.

[ref28] Gaertner S. L. Rust M. C. Dovidio J. F. Bachman B. A. Anastasio P. A. (1994). The contact hypothesis: the role of a common ingroup identity on reducing intergroup bias. Small Group Res. 25, 224–249. 10.1177/1046496494252005

[ref29] Galinsky A. D. Wang C. S. Ku G. (2008). Perspective-takers behave more stereotypically. J. Pers. Soc. Psychol. 95, 404–419. 10.1037/0022-3514.95.2.404, PMID: 18665710

[ref30] Geels F. W. (2011). The multi-level perspective on sustainability transitions: responses to seven criticisms. Environ. Innov. Soc. Transit. 1, 24–40. 10.1016/j.eist.2011.02.002

[ref31] Geels F. W. (2020). Micro-foundations of the multi-level perspective on socio-technical transitions: developing a multi-dimensional model of agency through crossovers between social constructivism, evolutionary economics and neo-institutional theory. Technol. Forecast. Soc. Change 152:119894. 10.1016/j.techfore.2019.119894

[ref32] Geels F. W. Schot J. (2007). Typology of sociotechnical transition pathways. Res. Policy 36, 399–417. 10.1016/j.respol.2007.01.003

[ref33] Gelfand M. J. Fulmer C. A. Severance L. (2011). “The psychology of negotiation and mediation” in APA handbook of industrial and organizational psychology, Vol. 3: Maintaining, expanding, and contracting the organization. ed. Zedeck S. (American Psychological Association), 495–554.

[ref34] Gillespie J. J. Thompson L. L. Loewenstein J. Gentner D. (1999). Lessons from analogical reasoning in the teaching of negotiation. Negot. J. 15, 363–371. 10.1111/j.1571-9979.1999.tb00734.x

[ref35] Hardisty D. J. Weber E. U. (2009). Discounting future green: money versus the environment. J. Exp. Psychol. Gen. 138, 329–340. 10.1037/a0016433, PMID: 19653793

[ref36] Hauser O. P. Rand D. G. Peysakhovich A. Nowak M. A. (2014). Cooperating with the future. Nature 511, 220–223. 10.1038/nature13530, PMID: 25008530

[ref37] Henderson M. D. Trope Y. Carnevale P. J. (2006). Negotiation from a near and distant time perspective. J. Pers. Soc. Psychol. 91, 712–729. 10.1037/0022-3514.91.4.712, PMID: 17014295 PMC3153434

[ref38] Herrnstein R. J. Prelec D. (1991). Melioration: a theory of distributed choice. J. Econ. Perspect. 5, 137–156. 10.1257/jep.5.3.137

[ref39] Hershfield H. E. (2011). Future self-continuity: how conceptions of the future self transform intertemporal choice. Ann. N. Y. Acad. Sci. 1235, 30–43. 10.1111/j.1749-6632.2011.06201.x, PMID: 22023566 PMC3764505

[ref40] Hsiang S. M. Burke M. Miguel E. (2013). Quantifying the influence of climate on human conflict. Science 341:1235367. 10.1126/science.1235367, PMID: 24031020

[ref41] Jacquet J. Hagel K. Hauert C. Marotzke J. Röhl T. Milinski M. (2013). Intra- and intergenerational discounting in the climate game. Nat. Clim. Chang. 3, 1025–1028. 10.1038/nclimate2024

[ref42] Jang D. Elfenbein H. A. Bottom W. P. (2018). More than a phase: form and features of a general theory of negotiation. Acad. Manag. Ann. 12, 318–356. 10.5465/annals.2016.0053

[ref43] Jehn K. A. (1995). A multimethod examination of the benefits and detriments of intragroup conflict. Adm. Sci. Q. 40:256. 10.2307/2393638

[ref44] Kamijo Y. Komiya A. Mifune N. Saijo T. (2017). Negotiating with the future: incorporating imaginary future generations into negotiations. Sustain. Sci. 12, 409–420. 10.1007/s11625-016-0419-8, PMID: 30147758 PMC6086238

[ref45] Kelley H. H. Thibaut J. W. (1978). Interpersonal relations: A theory of interdependence. New York, NY: Wiley.

[ref46] Kemp R. Loorbach D. Rotmans J. (2007). Transition management as a model for managing processes of co-evolution towards sustainable development. Int. J. Sustain. Dev. World Ecol. 14, 78–91. 10.1080/13504500709469709

[ref47] Kim J. Thompson L. Loewenstein J. (2020). Open for learning: encouraging generalization fosters knowledge transfer in negotiation. Negot. Confl. Manag. Res. 13, 3–23. 10.1111/ncmr.12163

[ref48] Levin K. Cashore B. Bernstein S. Auld G. (2012). Overcoming the tragedy of super wicked problems: constraining our future selves to ameliorate global climate change. Policy. Sci. 45, 123–152. 10.1007/s11077-012-9151-0

[ref49] Lewicki R. J. Litterer J. A. (1985). Negotiation. Homewood, IL: R.D. Irwin.

[ref50] Lewin K. (1948). Resolving social conflicts; selected papers on group dynamics. New York, NY: Harper.

[ref51] Loewenstein G. (1988). Frames of mind in intertemporal choice. Manag. Sci. 34, 200–214. 10.1287/mnsc.34.2.200

[ref52] Loewenstein G. (1996). Out of control: visceral influences on behavior. Organ. Behav. Hum. Decis. Process. 65, 272–292. 10.1006/obhd.1996.0028

[ref53] Loorbach D. (2010). Transition management for sustainable development: a prescriptive, complexity-based governance framework. Governance 23, 161–183. 10.1111/j.1468-0491.2009.01471.x

[ref54] Loschelder D. D. Trötschel R. (2010). Overcoming the competitiveness of an intergroup context: third-party intervention in intergroup negotiations. Group Process. Intergr. Relat. 13, 795–815. 10.1177/1368430210374482

[ref55] Mach K. J. Kraan C. M. Adger W. N. Buhaug H. Burke M. Fearon J. D.. (2019). Climate as a risk factor for armed conflict. Nature 571, 193–197. 10.1038/s41586-019-1300-6, PMID: 31189956

[ref56] Majer J. M. Loschelder D. D. Windolph L. J. Fischer D. (2018). How sustainability-related challenges can fuel conflict between organizations and external stakeholders: a social psychological perspective to master value differences, time horizons, and resource allocations. Umweltpsychol. 22, 53–70.

[ref57] Meadowcroft J. (2009). What about the politics? Sustainable development, transition management, and long term energy transitions. Policy. Sci. 42:323. 10.1007/s11077-009-9097-z

[ref58] Nadler J. Thompson L. Boven L. V. (2003). Learning negotiation skills: four models of knowledge creation and transfer. Manag. Sci. 49, 529–540. 10.1287/mnsc.49.4.529.14431

[ref59] Neale M. A. Bazerman M. H. (1985). The effects of framing and negotiator overconfidence on bargaining behaviors and outcomes. Acad. Manag. J. 28, 34–49. 10.2307/256060

[ref60] O’Connor K. M. De Dreu C. K. W. Schroth H. Barry B. Lituchy T. R. Bazerman M. H. (2002). What we want to do versus what we think we should do: an empirical investigation of intrapersonal conflict. J. Behav. Decis. Mak. 15, 403–418. 10.1002/bdm.426

[ref61] Okhuysen G. A. Galinsky A. D. Uptigrove T. A. (2003). Saving the worst for last: the effect of time horizon on the efficiency of negotiating benefits and burdens. Organ. Behav. Hum. Decis. Process. 91, 269–279. 10.1016/S0749-5978(03)00023-2

[ref62] Ornetzeder M. Rohracher H. (2013). Of solar collectors, wind power, and car sharing: comparing and understanding successful cases of grassroots innovations. Glob. Environ. Chang. 23, 856–867. 10.1016/j.gloenvcha.2012.12.007

[ref63] Ostrom E. (2009). A general framework for analyzing sustainability of social-ecological systems. Science 325, 419–422. 10.1126/science.1172133, PMID: 19628857

[ref64] Peters B. G. (2017). What is so wicked about wicked problems? A conceptual analysis and a research program. Polic. Soc. 36, 385–396. 10.1080/14494035.2017.1361633

[ref65] Pinkley R. L. Griffith T. L. Northcraft G. B. (1995). “Fixed pie” a la mode: information availability, information processing, and the negotiation of suboptimal agreements. Organ. Behav. Hum. Decis. Process. 62, 101–112. 10.1006/obhd.1995.1035

[ref66] Pruitt D. G. Carnevale P. J. (1993). Negotiation in social conflict. Belmont, CA: Thomson Brooks/Cole Publishing Co.

[ref67] Raiffa H. (1982). The art and science of negotiation. Cambridge, MA: Belknap Press of Harvard University Press.

[ref68] Raven R. P. Heiskanen E. Lovio R. Hodson M. Brohmann B. (2008). The contribution of local experiments and negotiation processes to field-level learning in emerging (niche) technologies: meta-analysis of 27 new energy projects in Europe. Bull. Sci. Technol. Soc. 28, 464–477. 10.1177/0270467608317523

[ref69] Read D. Loewenstein G. Rabin M. Keren G. Laibson D. (1999). “Choice bracketing” in Elicitation of preferences. eds. Fischhoff B. Manski C. F. (Dordrecht, Netherlands: Springer), 171–202.

[ref70] Rhoades J. A. Carnevale P. J. (1999). The behavioral context of strategic choice in negotiation: a test of the dual concern model 1. J. Appl. Soc. Psychol. 29, 1777–1802. 10.1111/j.1559-1816.1999.tb00152.x

[ref71] Ritov I. Drory A. (1996). Ambiguity and conflict management strategy. Int. J. Confl. Manag. 7, 139–155. 10.1108/eb022779

[ref72] Rittel H. W. Webber M. M. (1973). Dilemmas in a general theory of planning. Policy. Sci. 4, 155–169. 10.1007/BF01405730

[ref73] Rusbult C. E. Van Lange P. A. M. (1996). “Interdependence processes” in Social psychology: Handbook of basic principles eds. Higgins E. T. Kruglanski A. W. (New York: The Guilford Press), 564–596.

[ref74] Schelling T. C. (1958). The strategy of conflict. Prospectus for a reorientation of game theory. J. Confl. Resolut. 2, 203–264. 10.1177/002200275800200301

[ref75] Schelling T. C. (1984). Choice and consequence. Cambridge, MA: Harvard University Press.

[ref76] Schreuer A. Ornetzeder M. Rohracher H. (2010). Negotiating the local embedding of socio-technical experiments: a case study in fuel cell technology. Tech. Anal. Strat. Manag. 22, 729–743. 10.1080/09537325.2010.496286

[ref77] Schuster C. Majer J. M. Trötschel R. (2020). Whatever we negotiate is not what I like: how value-driven conflicts impact negotiation behaviors, outcomes, and subjective evaluations. J. Exp. Soc. Psychol. 90:103993. 10.1016/j.jesp.2020.103993

[ref78] Seyfang G. Haxeltine A. (2012). Growing grassroots innovations: exploring the role of community-based initiatives in governing sustainable energy transitions. Environ. Plan. Govern. Pol. 30, 381–400. 10.1068/c10222

[ref79] Sherif M. (1961). Intergroup conflict and cooperation: The robbers cave experiment. Vol. 10. OK: University Book Exchange Norman.

[ref80] Sherif M. Sherif C. W. (1953). Groups in harmony and tension; An integration of studies of intergroup relations. New York, NY: Harper & Brothers.

[ref81] Sherstyuk K. Tarui N. Ravago M.-L. V. Saijo T. (2016). Intergenerational games with dynamic externalities and climate change experiments. J. Assoc. Environ. Resour. Econ. 3, 247–281. 10.1086/684162

[ref82] Soman D. Ainslie G. Frederick S. Li X. Lynch J. Moreau P.. (2005). The psychology of intertemporal discounting: why are distant events valued differently from proximal ones? Mark. Lett. 16, 347–360. 10.1007/s11002-005-5897-x

[ref83] Sondeijker S. Geurts J. Rotmans J. Tukker A. (2006). Imagining sustainability: the added value of transition scenarios in transition management. Foresight 8, 15–30. 10.1108/14636680610703063

[ref84] Sunstein C. R. Reisch L. A. (2013). Green by default. Kyklos 66, 398–402. 10.1111/kykl.12028

[ref85] Tajfel H. (1981). Human groups and social categories: Studies in social psychology. Cambridge, MA: Cambridge University Press.

[ref86] Tajfel H. Turner J. C. (1979). “An integrative theory of intergroup conflict” in The social psychology of intergroup relations. eds. Austin W. G. Worchel S. (Monterey, CA: Brooks/Cole), 33–37.

[ref87] Tajfel H. Turner J. C. (1986). “The social identity theory of intergroup behavior” in Psychology of intergroup relations. eds. Worchel S. Austin W. G. (Chicago: Nelson-Hall), 7–24.

[ref88] Tavoni A. Dannenberg A. Kallis G. Loschel A. (2011). Inequality, communication, and the avoidance of disastrous climate change in a public goods game. Proc. Natl. Acad. Sci. U. S. A. 108, 11825–11829. 10.1073/pnas.1102493108, PMID: 21730154 PMC3141931

[ref89] Thompson L. DeHarpport T. (1994). Social judgment, feedback, and interpersonal learning in negotiation. Organ. Behav. Hum. Decis. Process. 58, 327–345. 10.1006/obhd.1994.1040

[ref90] Thompson L. Gonzalez R. (1997). “Environmental disputes: competition for scarce resources and clashing of values” in Environment, ethics, and behavior: The psychology of environmental valuation and degradation eds. Bazerman M. H. Messick D. M. Tenbrunsel A. E. Wade-Benzoni K. A. (San Francisco: The New Lexington Press/Jossey-Bass Publishers), 75–104.

[ref91] Thompson L. Hastie R. (1990). Social perception in negotiation. Organ. Behav. Hum. Decis. Process. 47, 98–123. 10.1016/0749-5978(90)90048-E

[ref92] Trötschel R. Hüffmeier J. Loschelder D. D. (2010). When yielding pieces of the pie is not a piece of cake: identity-based intergroup effects in negotiations. Group Process. Intergr. Relat. 13, 741–763. 10.1177/1368430210374608

[ref93] Trötschel R. Hüffmeier J. Loschelder D. D. Schwartz K. Gollwitzer P. M. (2011). Perspective taking as a means to overcome motivational barriers in negotiations: when putting oneself into the opponent’s shoes helps to walk toward agreements. J. Pers. Soc. Psychol. 101, 771–790. 10.1037/a0023801, PMID: 21728447

[ref94] Trötschel R. Loschelder D. D. Höhne B. P. Majer J. M. (2015). Procedural frames in negotiations: how offering my resources versus requesting yours impacts perception, behavior, and outcomes. J. Pers. Soc. Psychol. 108, 417–435. 10.1037/pspi0000009, PMID: 25751716

[ref95] Tsay C. J. Bazerman M. H. (2009). A decision-making perspective to negotiation: a review of the past and a look to the future. Negot. J. 25, 467–480. 10.1111/j.1571-9979.2009.00239.x

[ref96] Tuncel E. Mislin A. Kesebir S. Pinkley R. L. (2016). Agreement attraction and impasse aversion: reasons for selecting a poor deal over no deal at all. Psychol. Sci. 27, 312–321. 10.1177/0956797615619200, PMID: 26786822

[ref97] Turner J. C. Hogg M. A. Oakes P. J. Reicher S. D. Wetherell M. S. (1987). Rediscovering the social group: A self-categorization theory. Oxford, England: Basil Blackwell.

[ref98] Urminsky O. (2017). The role of psychological connectedness to the future self in decisions over time. Curr. Dir. Psychol. Sci. 26, 34–39. 10.1177/0963721416668810

[ref99] Van Boven L. Ehret P. J. Sherman D. K. (2018). Psychological barriers to bipartisan public support for climate policy. Perspect. Psychol. Sci. 13, 492–507. 10.1177/1745691617748966, PMID: 29961412

[ref100] Van der Gaast W. (2015). International climate negotiation conditions: Past and future. Groningen: University of Groningen, SOM research school.

[ref101] Van Lange P. A. M. Balliet D. (2015). “Interdependence theory” in APA handbook of personality and social psychology, Vol. 3: Interpersonal relations. eds. Mikulincer M. Shaver P. R. Simpson J. A. Dovidio J. F. (Washington, DC: American Psychological Association), 65–92.

[ref102] von Wirth T. Gislason L. Seidl R. (2018). Distributed energy systems on a neighborhood scale: reviewing drivers of and barriers to social acceptance. Renew. Sust. Energ. Rev. 82, 2618–2628. 10.1016/j.rser.2017.09.086

[ref103] Wade-Benzoni K. A. (2008). Maple trees and weeping willows: the role of time, uncertainty, and affinity in intergenerational decisions. Negot. Confl. Manag. Res. 1, 220–245. 10.1111/j.1750-4716.2008.00014.x

[ref104] Wade-Benzoni K. A. Hernandez M. Medvec V. Messick D. (2008). In fairness to future generations: the role of egocentrism, uncertainty, power, and stewardship in judgments of intergenerational allocations. J. Exp. Soc. Psychol. 44, 233–245. 10.1016/j.jesp.2007.04.004

[ref105] Wade-Benzoni K. A. Tost L. P. (2009). The egoism and altruism of intergenerational behavior. Pers. Soc. Psychol. Rev. 13, 165–193. 10.1177/1088868309339317, PMID: 19571118

[ref106] Wade-Benzoni K. A. Tost L. P. Hernandez M. Larrick R. P. (2012). It’s only a matter of time: death, legacies, and intergenerational decisions. Psychol. Sci. 23, 704–709. 10.1177/0956797612443967, PMID: 22692338

[ref107] Weber E. U. (2017). Breaking cognitive barriers to a sustainable future. Nat. Hum. Behav. 1:13. 10.1038/s41562-016-0013

[ref108] Weber E. U. Johnson E. J. (2016). “Can we think of the future? Cognitive barriers to future-oriented thinking” in Global cooperation and the human factor eds. Messner D. Weinlich S. (New York, NY: Routledge), 139–154.

[ref111] Weber E. U. Johnson E. J. Milch K. F. Chang H. Brodscholl J. C. Goldstein D. G. (2007). Asymmetric discounting in intertemporal choice: a query-theory account. Psychol. Sci. 18, 516–523. 10.1111/j.1467-9280.2007.01932.x 17576265

[ref109] Wildschut T. Insko C. A. (2007). Explanations of interindividual—intergroup discontinuity: a review of the evidence. Eur. Rev. Soc. Psychol. 18, 175–211. 10.1080/10463280701676543

[ref110] Yoeli E. Hoffman M. Rand D. G. Nowak M. A. (2013). Powering up with indirect reciprocity in a large-scale field experiment. Proc. Natl. Acad. Sci. U. S. A. 110, 10424–10429. 10.1073/pnas.1301210110, PMID: 23754399 PMC3690615

